# MYB2 Is Important for Tapetal PCD and Pollen Development by Directly Activating Protease Expression in *Arabidopsis*

**DOI:** 10.3390/ijms23073563

**Published:** 2022-03-24

**Authors:** Xiaorui Guo, Lihong Li, Xiatong Liu, Chong Zhang, Xiaoyun Yao, Zhili Xun, Zhijing Zhao, Wenwen Yan, Yirong Zou, Di Liu, Hui Li, Hai Lu

**Affiliations:** 1 Beijing Advanced Innovation Center for Tree Breeding by Molecular Design, Beijing Forestry University, Beijing 100083, China; guoxiaorui0404@163.com (X.G.); luhai1974@bjfu.edu.cn (H.L.); 2 National Engineering Laboratory for Tree Breeding, College of Biological Sciences and Biotechnology, Beijing Forestry University, Beijing 100083, China; liudi@bjfu.edu.cn; 3 The Tree and Ornamental Plant Breeding and Biotechnology Laboratory of National Forestry and Grassland Administration, College of Biological Sciences and Biotechnology, Beijing Forestry University, Beijing 100083, China; lilihong0215@163.com (L.L.); summertong621@163.com (X.L.); chongzhang@bjfu.edu.cn (C.Z.); yaoxiaoyun0102@163.com (X.Y.); zhili20052@126.com (Z.X.); zzjkate310@163.com (Z.Z.); Yan88963@163.com (W.Y.); zouyr706@163.com (Y.Z.)

**Keywords:** transcription factor, proteases, tapetal PCD, pollen development, regulatory cascade, *Arabidopsis thaliana*

## Abstract

Tapetal programmed cell death (PCD) is a complex biological process that plays an important role in pollen formation and reproduction. Here, we identified the *MYB2* transcription factor expressed in the tapetum from stage 5 to stage 11 that was essential for tapetal PCD and pollen development in *Arabidopsis* *thaliana*. Downregulation of *MYB2* retarded tapetal degeneration, produced defective pollen, and decreased pollen vitality. EMSA and transcriptional activation analysis revealed that MYB2 acted as an upstream activator and directly regulated expression of the proteases *CEP1* and *βVPE*. The expression of these proteases was lower in the buds of the *myb2* mutant. Overexpression of either/both *CEP1* or/and *βVPE* proteases partially recover pollen vitality in the *myb2* background. Taken together, our results revealed that *MYB2* regulates tapetal PCD and pollen development by directly activating expression of the proteases *CEP1* and *βVPE*. Thus, a transcription factor/proteases regulatory and activated cascade was established for tapetal PCD during another development in *Arabidopsis* *thaliana*. Highlight: *MYB2* is involved in tapetal PCD and pollen development by directly regulating expression of the protease *CEP1* and *βVPE* and establishes a transcription factor/proteases regulatory and activated cascade.

## 1. Introduction

The anther is an essential reproductive organ in flower plants with four similarly structured lobes, consisting of the epidermis, the endothecium, the middle layer, and the tapetum from exterior to interior. Microspores, which complete two mitoses to form mature pollen, are surrounded by the anther lobes. The tapetum provides enzymes, nutrients, lipids, and polysaccharides for microspores development and pollen formation via tapetum cell degeneration. Therefore, the process of tapetal degradation is critical for pollen development and abnormal tapetal degeneration results in defective pollen or male sterility [1,2,3].

Tapetal degeneration is triggered by programmed cell death (PCD) [3]. In plants, members of proteases participate in PCD during diverse biological processes [4,5,6,7,8]. Several proteases are associated with tapetal PCD and pollen formation. An *Arachis diogoi* cysteine protease (*AdCP*) is expressed under the tapetum-specific promoter (TA29) in tobacco and *Brassica juncea* caused male sterility [9,10]. The cysteine protease NtCP56 is essential for pollen grain development in *Nicotiana tabacum L.* [11]. The *Arabidopsis* anther-specific cysteine protease CP51 is involved in tapetal degradation and formation of pollen exine [12]. *BnaC.CP20.1*, identified from *Brassica napus*, refers to tapetal degeneration and pollen wall formation [13]. Previous studies in our lab reported that an *Arabidopsis* papain-like cysteine protease CEP1 is an irreplaceable executor involved in tapetum cell degeneration and pollen formation [14]. We also identified the vacuolar processing enzyme βVPE, which is indirectly involved in tapetal PCD and pollen development by directly activating the maturation of cysteine proteases, including CEP1, RD19A, and RD19C [15]. The loss or ectopic expression of these proteases leads to abnormal tapetal PCD and defective pollen. *CEP1* was expressed in anther from stage 5 to stage 11. The *cep1* mutant showed rerarded tapetal PCD and decreased pollen viability [14]. *β VPE* was expressed in the anther of stage 5–8, and *βvpe* mutant exhibited a similar defect anther phenotype to the *cep1* mutant [15]. These findings suggest that precise expression of proteases is necessary for their function during tapetal degeneration and pollen development.

A number of genes encode putative transcription factors that are involved in tapetal and pollen development, including *Arabidopsis DYSFUNCTIONAL TAPETUM1* (*DYT1*) [16], *Defective in Tapetal Development and Function1* (*TDF1*) [17], *ABORTED MICROSPORES* (*AMS*) [18,19], *MALE STEILITY1* (*MS1*) [20], *MYB80* [21] and rice *OsTDF1* [22], *OsMS188* [23], *Undeveloped Tapetum1* (*UDT1*) [24], *ETERNAL TAPETUM1* (*EAT1*) [25], and *PERSISTANT TAPETAL CELL1/2* (*PTC1/2*) [26,27]. Several proteases are directly or indirectly regulated by these transcription factors [7]. For example, *UNDEAD* encodes an *Arabidopsis* A1 aspartic protease that is directly regulated by *MYB80* via the binding site on the *UNDEAD* promoter sequence. *MYB80* regulates the time of tapetal PCD by regulating the expression of *UNDEAD* [28]. Rice UDT1 may affects tapetal PCD by regulating expression of the aspartyl proteases *OsAP67* and *OsAP38* [24]. The rice transcription factor EAT1 promotes tapetum cell death by directly regulating the expression of two aspartic proteases *OsAP25* and *OsAP37* [25]. In addition, the expression of several *Arabidopsis* proteases such as *RD19A*, *RD19C*, and *RD21A* changes in varying levels in *dyt1*, *ams*, and *ms1* mutants and the rice proteases *OsCP1* and *OsAP25* are reduced in *ptc1/ptc2* mutants [15,26,27].

Numerous other undiscovered transcription factors involved in tapetal PCD and the regulatory mechanisms between these transcription factors and proteases in tapetal PCD and pollen development require further validation. Here, we identified a key tapetal PCD regulator *MYB2* (AT2G47190) and investigated its function in anther development. The partial male sterility in T-DNA insertion of *MYB2* is similar to the phenotype of *cep1* and *βvpe* mutants, and cis-elements prediction indicated that MYB2 cis-elements existed in the *CEP1* and *βVPE* promoter. In this study, we found that MYB2 directly bound to the *CEP1* and *βVPE* promoter and regulated *CEP1* and *βVPE* expression, thus involved in tapetal PCD and pollen development. These transcription factors/proteases genetic cascade extends the understanding about tapetal PCD regulation.

## 2. Results

### 2.1. Phenotypic Characterization of myb2 Mutant

**Figure 1:**
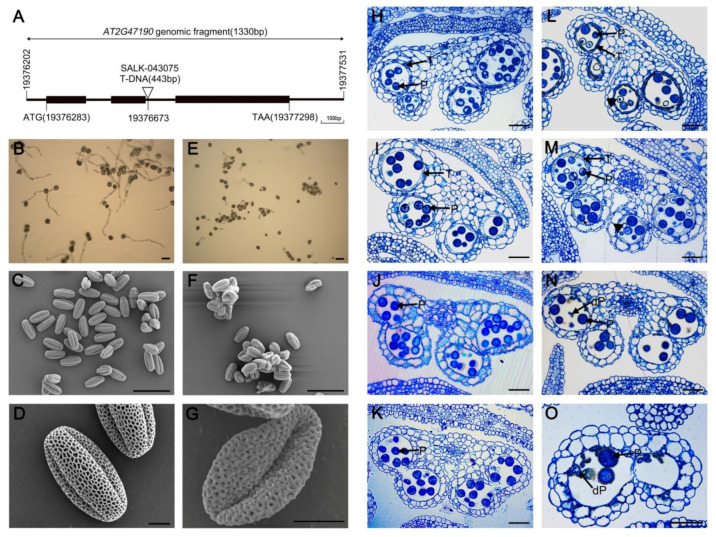
The phenotype of Arabidopsis *myb2* mutant plants. (**A**) SALK_043075 insertion positions in *AT2G47190*. (**B**,**E**) Germination rate of wild-type and *myb2* mutant pollen. (**B**) wild type; (**E**) *myb2* mutant. Bar = 50 μM. (**C**–**G**) Scanning electron microscopy of wild type pollen and *myb2* mutant pollen. (**C**,**D**) wild type pollen; (**F**,**G**) *myb2* mutant pollen. (**C**,**F**) Bar = 50 μM; (**D**,**G**) Bar = 5 μM. (**H**–**O**) Anther development in the wild type and *myb2* mutant. (**H**–**K**) wild-type anther. (**L**–**O**) *myb2* mutant anther. (**H**,**L**) stage 10; (**I**,**M**) early stage 11; (**J**,**N**) late stage 11; (**K**,**O**) stage 12. Bar = 50 μM. P, pollen; T, tapetum; dP, defective pollen.

Previous studies have shown that *MYB2* transcripts were expressed in anthers, but their function is unknown [29]. To analyze the function of *MYB2* during another development in *Arabidopsis*, we obtained a T-DNA insertion mutant line (SALK_043075) from the Arabidopsis Biological Resource Center (ABRC). The T-DNA was inserted into the second intron of *MYB2* in the mutant line (Figure 1A). Homozygous T-DNA insertion mutants were used for further analysis. The *myb2* mutant plants displayed a normal phenotype during vegetative development compared with wild-type plants. However, the germination rate of the pollen grains in vitro decreased significantly in *myb2* (29.55% ± 2.10) compared with that in the wild type (87.50% ± 1.68) (Figure 1B,E). A scanning electron microscopy (SEM) examination revealed that wild-type mature pollen grains were plump and uniformly spheroid and had finely reticulate ornamentation on their surface (Figure 1C,D). In contrast, the abnormal pollen grains in the *myb2* mutants had shrunk, formed irregular clumps, and the surface was deformed without the regularly reticulate ornamentation (Figure 1F,G). These results suggested that the loss of function of *MYB2* markedly influenced on pollen development and vitality.

Microscopic observations of semi-thin anther sections were made to further clarify the anther development in the wild-type and mutants. The development of *Arabidopsis* anthers is divided into 14 stages based on morphological landmarks that correspond to cellular events visible under a microscope [30]. Tapetal cells released stained materials into the anther locules in the wild type at stage 10, but little stained material was released from the *myb2* mutant tapetal cells (Figure 1H,L). At early stage 11, the tapetal cells were almost degraded and had a few remnants in wild type. However, the tapetal cells did not clearly degenerate and most remnants still remained in the *myb2* mutants (Figure 1I,M). The wild-type anther was filled with well-developed pollen grains from stage 10 to stage 11 (Figure 1H–J). In contrast, some *myb2* mutant pollen grains were still vacuolated (Figure 1L–N). The tapetal cells were completely degraded and the mature pollen grains were formed in the wild type at stage 12 (Figure 1K). Some pollen grains were defective and shrunken in the *myb2* mutants during the same stage (Figure 1O). These results showed that anther maturation was abnormal in the *myb2* mutants, particularly tapetal degeneration and pollen development.

### 2.2. Tapetal PCD Was Retarded in myb2 Mutant

**Figure 2:**
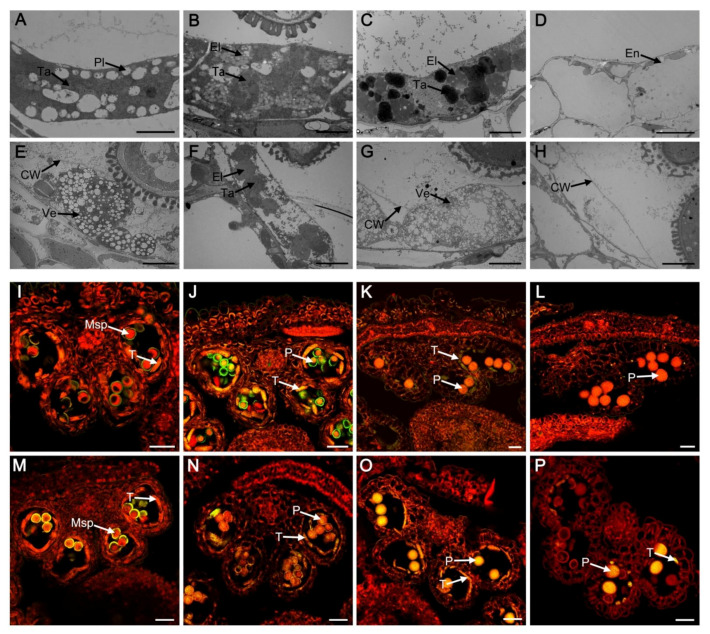
The tapetal development of wild-type and *myb2* mutant. (**A**–**H**) The taptum of anther at different developmental stages were detected by Transmission Electron Microscopy in wild-type and *myb2* mutant. (**A**–**D**) wild type; (**E**–**H**) *myb2* mutant. Bar = 5 μM. (**I**–**P**) The nuclear DNA fragmentation of anther using the TUNEL assay at different developmental stages were compared in the wild-type and *myb2* mutant. Nuclei were stained with propidium iodide indicated by the red fluorescence, and the yellow fluorescence is TUNEL positive signal. (**I**–**L**) wild type; (**M**–**P**) *myb2* mutant. Bar = 25 μM. (**A**,**E**), (**I**,**M**) stage 9; (**B**,**F**,**J**,**N**) stage 10; (**C**,**G**,**K**,**O**) stage 11; (**D**,**H**,**L**,**P**) stage 12. CW, cell wall; El, elaioplast; En, endothecium; Msp, microspore P, pollen; Pl, plastid; T, tapetum; Ta, tapetosome; Ve, vesicle.

To further investigate the differences in tapetal development between the wild type and the mutant lines, transmission electron microscopy (TEM) was performed. The tapetal wall degraded completely at stage 9 in the wild type. The plastids and tapetosome contained electron transparent deposits and lipid material was discovered in the tapetal cells (Figure 2A). In contrast, the tapetal wall remained intact in the *myb2* mutant and tapetal cells showed few tapetosomes and formed secretory plastids and vesicles (Figure 2E). The elaioplasts which were transformed from plastids and tapetosomes increased clearly in wild-type tapetal cells during stage 10 (Figure 2B). Fewer tapetosomes and elaioplasts were found in the *myb2* mutant than in the wild type (Figure 2F). Wild-type tapetal cells were filled with tapetosomes and elaioplasts and much osmiophilic material was released continuously into locules at stage 11 (Figure 2C). In contrast, the tapetal cells were spongy due to the presence of numerous vesicles and no obvious tapetosomes or elaioplasts were observed, resulting in little osmiophilic material being released in *myb2* mutants (Figure 2G). The tapetal cells were completely degenerated in the wild type at stage 12 (Figure 2D). The undegenerated tapetal wall remained in the anthers of *myb2* mutant. (Figure 2H). These results indicated that degeneration of the tapetal wall was defective, and the formation of secretory organelles decreased distinctly in the *myb2* mutant.

The terminal deoxynucleotidyl transferase-mediated dUTP nick-end labeling (TUNEL) assay is often used to assess cleavage of DNA (a characteristic of PCD). We further detected tapetal PCD of the wild type and mutants using the TUNEL assay at different developmental stages. TUNEL- positive signals were not different between the wild type and the *myb2* mutant at stage 9 (Figure 2I,M). Strong TUNEL-positive signals were detected in the wild-type tapetal cells at stage 10, indicating that tapetal cells were degenerating during this stage (Figure 2J). TUNEL-positive signals were weaker in the *myb2* tapetal cells at the same stage compared with the wild type (Figure 2N). The wild-type tapetal cells reached the end of PCD at stage 11 and TUNEL-positive signals decreased significantly (Figure 2K). However, the yellow signals were obvious in tapetal cells from the *myb2* mutant (Figure 2O). No TUNEL-positive signals were observed in the wild type at stage 12 due to complete degeneration of the tapetal cells (Figure 2L). In contrast, a few TUNEL-positive signals were present in the tapetal cell remnants of the *myb2* mutant (Figure 2P). Taken together, these results indicated that tapetal cell PCD was retarded in the *myb2* mutant.

### 2.3. Abnormal Pollen Development in the myb2 Mutant

**Figure 3:**
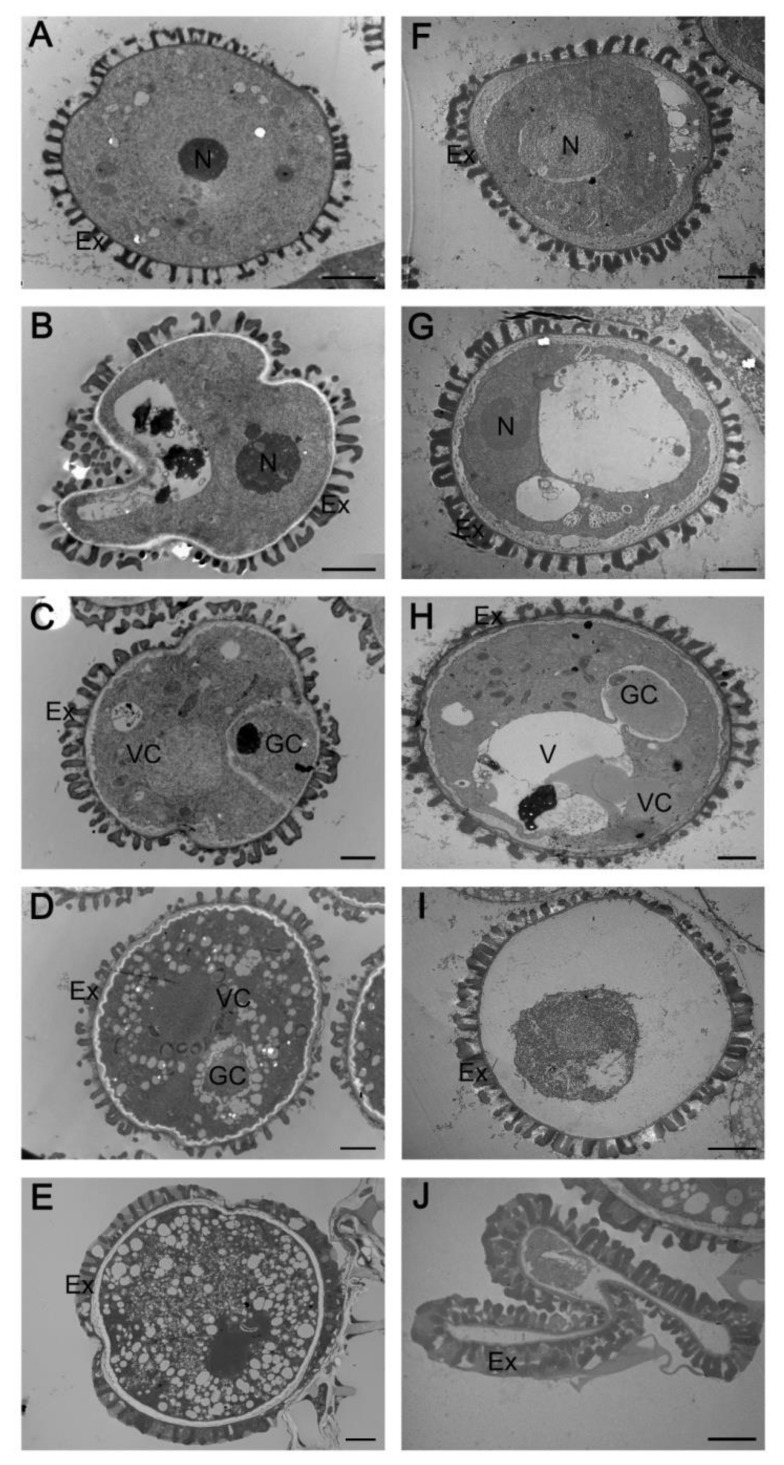
Transmission electron micrographs of microspores from the wild type and *myb2* mutant. Microspores at different developmental stages in the wild type (**A**–**E**) and *myb2* mutant (**F**–**J**). (**A**,**F**) stage 8; (**B**) and (**G**) stage 9; (**C**,**H**) stage 10; (**D**,**I**) stage 11; (**E**,**J**) stage 12. Bar = 2 μM. Ex, exine; GC, generative cell; N, nucleus; V, vacuole; VC, vegetative cell.

Abnormal tapetal PCD affects pollen development. We used TEM to follow pollen development in the *myb2* mutant and the wild type. No differences in microspore structure were observed between the *myb2* mutant and the wild type at stage 8 or stage 9 (Figure 3A,B,F,G). No significant vacuole was observed in the vegetative cell and the orderly microspore exine structure appeared in wild-type pollen at stage 10 (Figure 3C). However, *myb2* mutant pollen contained vacuoles and the exine was irregular and sparse at the same stage (Figure 3H). Immature pollen underwent enlargement of the cytoplasm and numerous oil bodies filled the pollen grain during stage 11. Generative cells moved away from the edge of the pollen grain in the wild type (Figure 3D). Development of the pollen cytoplasm was incomplete in the *myb2* mutant with an inconspicuous nucleolus and no generative cell (Figure 3I). The typical pollen structure was completely established during stage 12, with regular exine in the wild type (Figure 3E). However, the pollen shrank because of the insufficient filling of the cytoplasm and the exine was coarse in the *myb2* mutant (Figure 3J). These results show that pollen maturation was defective in the *myb2* mutant.

### 2.4. Complementation Analysis

**Figure 4:**
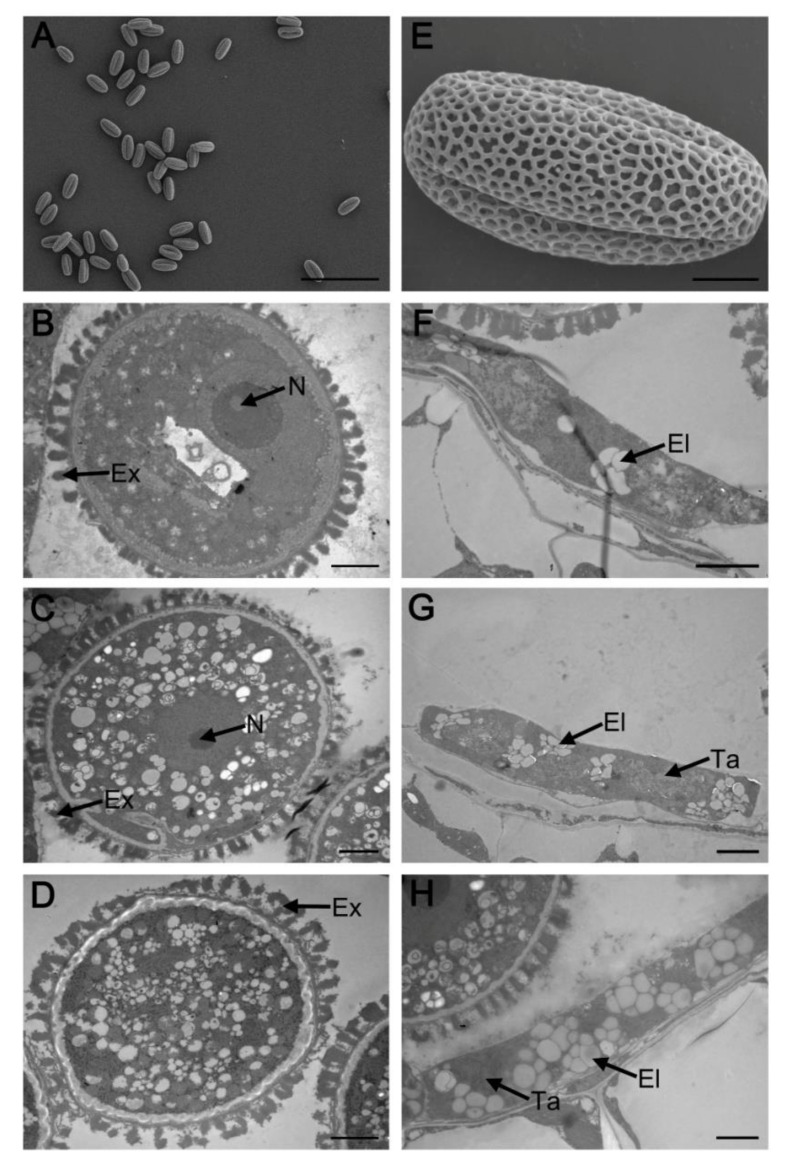
Complementation of the *myb2* mutants by *MYB2* genomic sequence. (**A**,**E**) Scanning electron microscopy of mature pollen grains in complementation lines. (**A**) Bar = 100 μM; (**E**) Bar = 5 μM. (**B**–**D**) Transmission electron micrographs of the microspore development in complementation lines. (**B**) stage 8; (**C**) stage 11; (**D**) stage 12. Bar = 2 μM. (**F**–**H**) Transmission electron micrographs of tapetum development in complementation lines. (**F**) early stage 10; (**G**) late stage 10; (**H**) stage 11. Bar = 2 μM.El, elaioplast; Ex, exine; N, nucleus; Ta, tapetosome.

We performed a functional complementation experiment to determine whether the *myb2* mutant phenotype was due to T-DNA insertion of *MYB2*. A 2666 bp genomic fragment including the promoter and the *MYB2* genomic sequence was cloned into the pCAMBIA1301 vector and transformed into *myb2* mutant plants. The regular reticulated surface was observed on the transgenic pollen grains by SEM (Figure 4A,E). TEM revealed normal pollen development and pollen exine structure in the complementation lines (Figure 4B,D). These results indicated that *MYB2* complementation in the *myb2* mutant restored pollen formation. In addition, tapetal degeneration was the same as the wild type, showing complete degradation of the tapetal wall and normal formation of the elaioplasts and tapetosomes (Figure 4F–H). Therefore, the successful rescue of tapetal degeneration and pollen development in the complementation lines indicated that the T-DNA insertion of *MYB2* was responsible for the mutant phenotype.

### 2.5. Spatio-Temporal Expression Pattern of MYB2

**Figure 5:**
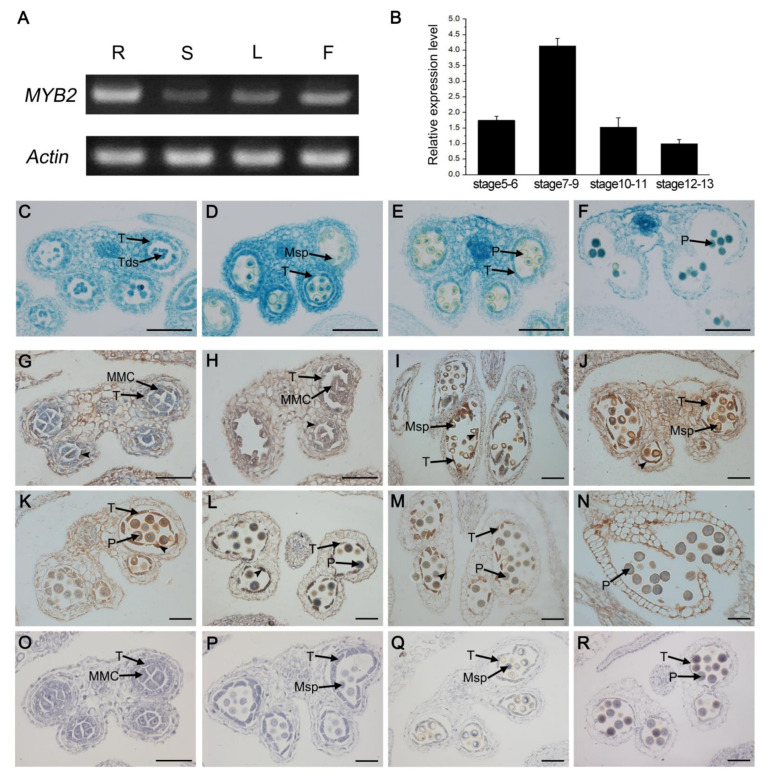
The gene expression pattern of *MYB2*. (**A**) *MYB2* expression analyses by RT-PCR. F, flower; L, leaf; R, root; S, stem. (**B**) RT-qPCR of *MYB2* expression in wild-type bud tissues at different developmental stages. Bars represent standard deviations. The expression of *MYB2* in stage 12–13 was set as 1. (**C**–**F**) Histochemical assay for GUS activity harboring the *MYB2* promoter-GUS fusion in anther at different stages. (**C**) stage 7; (**D**) stage 9; (**E**) stage 10; (**F**) stage 13. Bar = 20 μM. (**G**–**R**) In situ RNA hybridization analyses of *MYB2* expression pattern in wild-type anthers. (**G**) stage 5; (**H**) stage 6; (**I**) stage 8; (**J**) stage 9; (**K**) early stage 10; (**L**) late stage 10; (**M**) stage 11; (**N**) stage 13; (**O**) negative controls at stage 5; (**P**) negative controls at stage 8; (**Q**) negative controls at stage 9; (**R**) negative controls at stage 11. Bar = 50 μM. MMC, microspore mother cell; Msp, microspore; P, pollen; T, tapetum; Tds, tetrads.

We investigated the *MYB2* expression characteristics to further determine the relationship between the function and expression of *MYB2*. We performed RT-PCR analysis with the total RNA extracted from various organs, including roots, stems, leaves, and buds. *MYB2* was highly expressed in buds and roots but expressed at relatively low levels in leaves and stems (Figure 5A). We also evaluated the *MYB2* expression levels in buds at different developmental stages by RT-qPCR. *MYB2* expression in buds was already detectable at stages 5–6, reached the maximum at stages 7–9, and then decreased to a low level at stages 10–13 (Figure 5B). To confirm the *MYB2* expression pattern in anthers, we examined the spatiotemporal expression using a GUS reporter assay and in situ hybridization. First, we transformed wild-type *Arabidopsis* with a GUS (β-glucuronidase) reporter driven by the 1048 bp *MYB2* promoter (Pro*MYB2*: GUS). A transverse section analysis of the GUS-stained buds indicated that GUS signals were appeared in the epidermis, endothecium, middle layer, and tapetum of the anther at stage 7, became stronger and reached the maximum at stage 9, and then declined from stages 10–13 when the tapetum degraded (Figure 5C–F). In situ RNA hybridization was performed at different stages during anther development. The *MYB2* transcripts were detected in tapetal cells and other somatic tissues including epidermis, endothecium, and middle layer at late stage 5 (Figure 5G). *MYB2* expression predominantly increased in the tapetum during stage 6–9, but was at relatively low levels in other somatic tissues (Figure 5H–J). *MYB2* expression in the tapetum decreased gradually from stage 10 to 13 along with tapetal degeneration and was undetectable until the end of tapetal degeneration (Figure 5K–N). Taken together, the spatiotemporal expression pattern of *MYB2* (expressed in tapetum during stage 5 to 11) was consistent with its function in tapetal PCD.

### 2.6. MYB2 Directly Binds to Cysteine Proteases CEP1 and βVPE Promoter and Activates Their Expression

**Figure 6:**
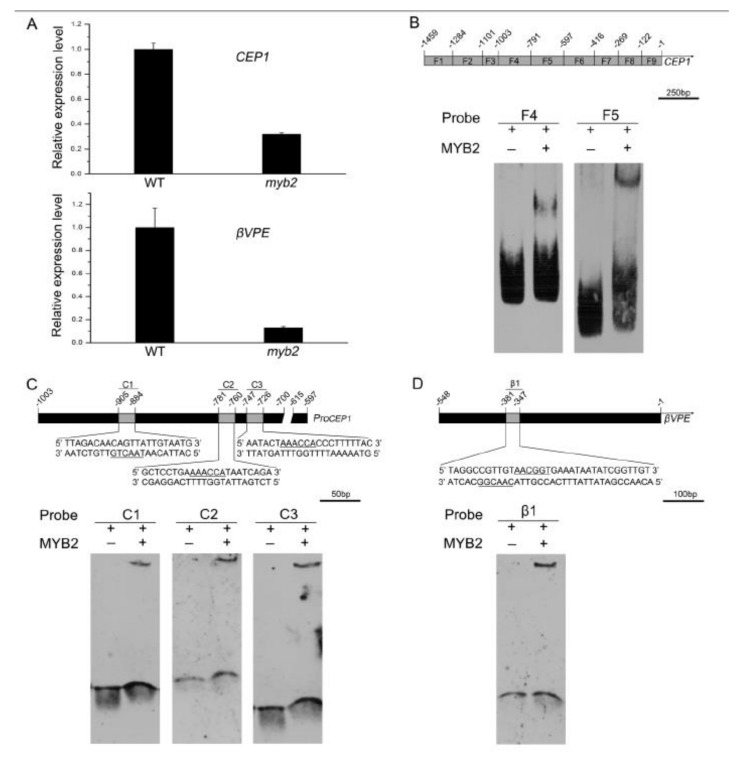

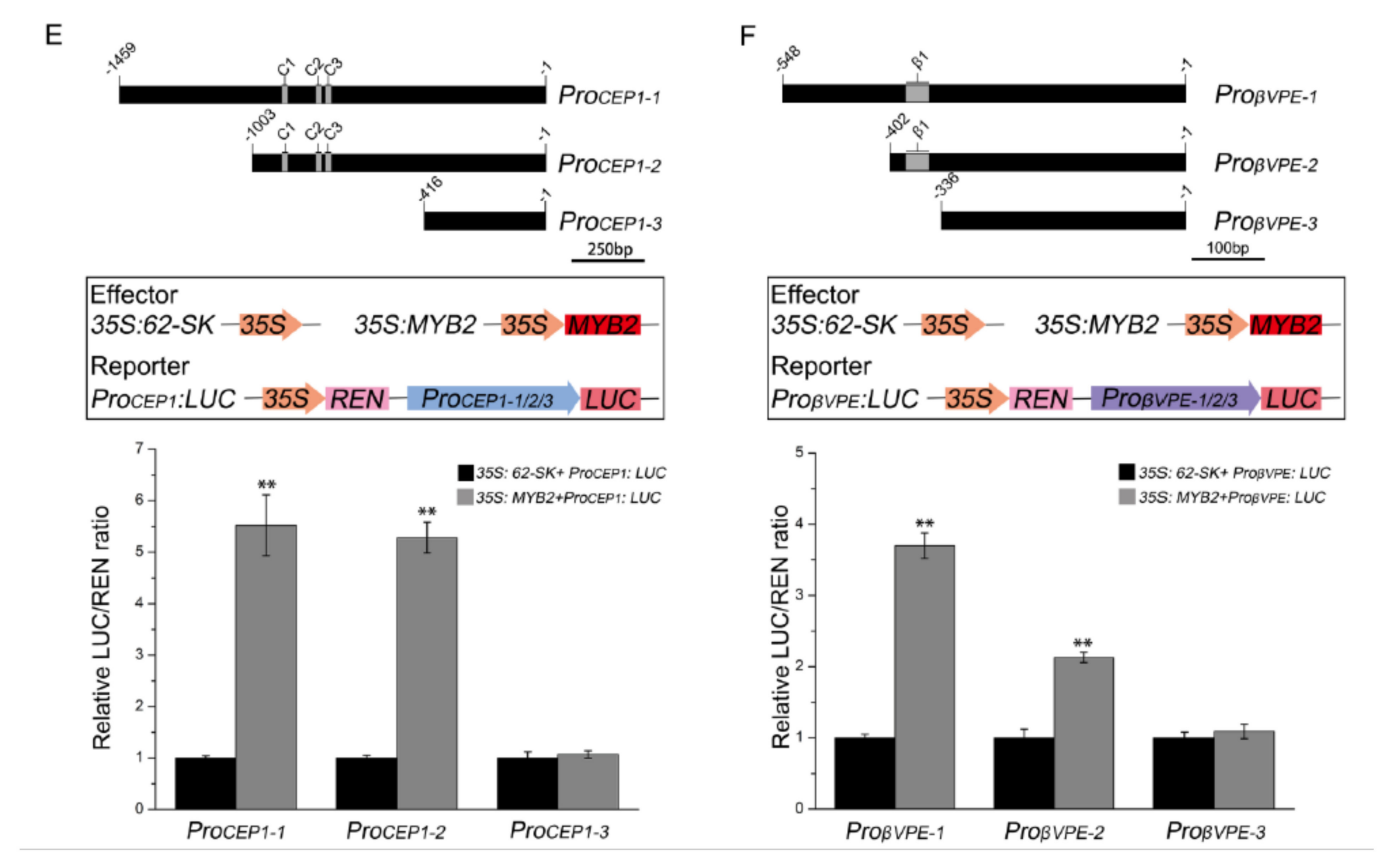
*MYB2* regulates the expression of *CEP1* and *βVPE*. (**A**) RT-qPCR of *CEP1* and *βVPE* expression in wild-type and *myb2* mutant bud tissues. *CEP1* and *βVPE* expression in wild type was set as 1. (**B**) EMSA showing that HIS-MYB2 binds to the −597 to −791 region and the −792 to −1003 region of *CEP1* promoter. (**C**) EMSA showing that HIS-MYB2 binds to the short probes of the *CEP1* promoter. (**D**) EMSA showing that HIS-MYB2 binds to the short probe of the *βVPE* promoter. (**E**) Transcriptional activation analysis of the full-length and truncated *CEP1* promoter. (**F**) Transcriptional activation analysis of the full-length and truncated *βVPE* promoter. Transient expression assay of relative luciferase activity, shown a ratio of LUC to REN in *Nicotiana benthamiana* leaves. 35S: *REN* was applied as an internal control. The ratio of LUC to REN of effector *35S: 62-SK* was set to 1. ** indicated *p* ≤ 0.01.

*MYB2* was observed in tapetal cells at stages 5–11 (when *CEP1* and *βVPE* were expressed) during anther development. The anther phenotype of the *myb2* mutant (retarded tapetal degeneration and defective pollen) was partly similar to *cep1* and *βvpe* [14,15]. We speculate that *MYB2*, *CEP1*, and *βVPE* function in a common regulatory pathway for tapetal PCD. To test this possibility, we examined the expression of *CEP1* and *βVPE* in the *myb2* mutant by RT-qPCR. As expected, *CEP1* and *βVPE* expression was significantly downregulated in the *myb2* mutant, confirming that *CEP1* and *βVPE* are downstream of *MYB2* (Figure 6A). An electrophoretic mobility shift assay (EMSA) was implemented to further investigate whether *CEP1* and *βVPE* were MYB2 target genes. According to the PLACE database (https://www.dna.affrc.go.jp/PLACE/, 15 March 2022), several putative recognition sites for MYB-related proteins were found in the 1459-bp *CEP1* promoter sequence. Previous studies have reported that MYB2 was predicted regulating target genes by the A/TAACCA or C/TAACG/TG motifs [31]. To determine the proper recognition region for MYB2, the *CEP1* promoter was divided into nine fragments containing different MYB-related recognition sites (Supplemental Figure S1). The purified HIS-MYB2 protein and *CEP1* promoter probes were used for EMSA assays ( Supplemental Figure S2). The EMSA results implied that key elements of MYB2 binding were located in the −597 to −791 region with two AAACCA motifs and the −792 to −1003 region with one TAACTG motif of the *CEP1* promoter (Figure 6B). Three short CEP1 promoter probes containing the TAACTG motif or AAACCA motifs were generated for the EMSA assay. The EMSA results show that the MYB2 protein bound to *CEP1* promoter fragments with AAACCA or TAACTG motif, which was consistent with the previous prediction (Figure 6C). A transcriptional activation analysis was performed in tobacco leaves to verify whether MYB2 activated the expression of *CEP1* by MYB2 binding sites. The full-length *CEP1* promoter and truncated version containing or lacking MYB2 binding regions were inserted into the pGreenII 0800-LUC to generate reporter constructs, respectively (Figure 6E). The relative luciferase activity data established that LUC activity driven by the full-length *CEP1* promoter and truncated promoter containing the MYB2 binding sites were induced significantly by MYB2 (Figure 6E). However, MYB2 did not induce the activity of LUC driven by the truncated *CEP1* promoter without the MYB2 binding sites (Figure 6E).

Moreover, a sequence analysis of the 548-bp *βVPE* promoter using the PLACE database revealed the presence of two contiguous putative MYB2-binding motifs (TAACGG and CAACGG). The EMSA results indicated that MYB2 bound to the labeled *βVPE* promoter fragment containing TAACGG and CAACGG (Figure 6D). The full-length *βVPE* promoter and truncated version containing or lacking the MYB2 binding regions were inserted into the reporter construct for transcriptional activation analysis (Figure 6F). The relative luciferase activity data indicated that MYB2 induced the LUC activity driven by the *βVPE* promoter containing the MYB2-binding sites but was not induced in the absence of the MYB2-binding sites (Figure 6F). Taken together, these results revealed that MYB2 directly binds to the *CEP1* and *βVPE* promoters by MYB2 binding sites and activated their expression.

### 2.7. The Deficiency of Cysteine Proteases Is Responsible for myb2 Mutant Phenotype

**Figure 7:**
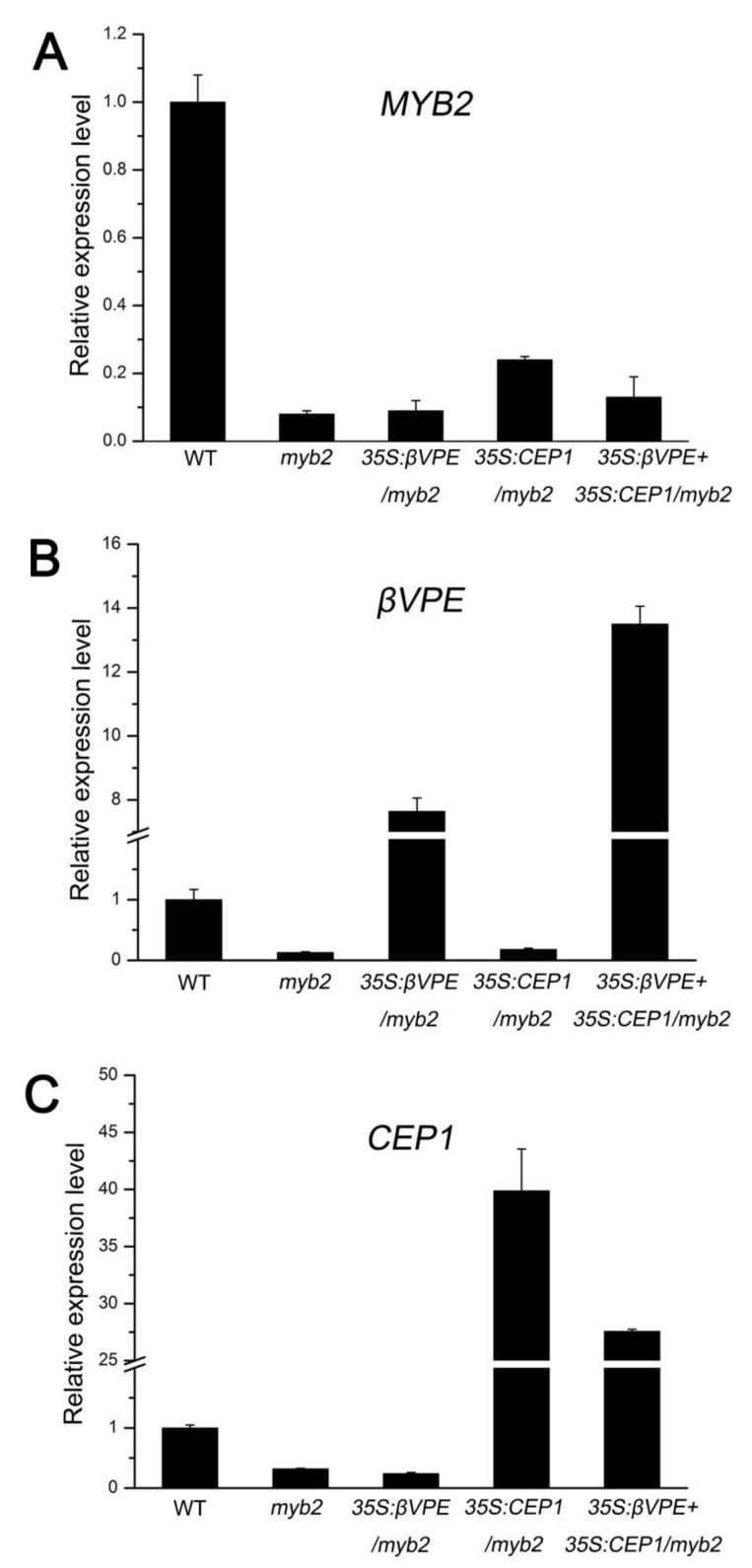
The complementation of protease in *myb2* background. (**A**) RT-qPCR of *MYB2* expression in bud tissues from the different lines. *MYB2* expression in the wild type was selected as 1. (**B**) RT-qPCR of *βVPE* expression in bud tissues from different lines. *βVPE* expression in the wild type was selected as 1. (**C**) RT-qPCR of *CEP1* expression in bud tissues from different lines. *CEP1* expression in the wild type was selected as 1.

To confirm whether downregulation of *CEP1* and *βVPE* in the *myb2* mutant was responsible for the *myb2* phenotype, we overexpressed the *CEP1* and *βVPE*, *CEP1*, and *βVPE* genes in the *myb2* mutant background to establish complementation transgenic lines. The RT-qPCR results showed that the *CEP1* or *βVPE* transcription level decreased in all *myb2* mutant background lines compared with the wild type (Figure 7A–C). However, *CEP1* expression increased significantly in 35S: *CEP1*/*myb2* (107.78% ± 9.91) and 35S: *CEP1* + 35S: *βVPE*/*myb2* (74.52% ± 0.43) compared with *myb2* mutant (Figure 7C). *βVPE* expression increased significantly in 35S: *βVPE*/*myb2* (58.82 ± 3.24%) and 35S: *CEP1* + 35S: *βVPE*/*myb2* (103.85 ± 4.29%) compared with *myb2* mutant (Figure 7B). We observed the morphology of the pollen grains by SEM and determined the pollen germination rate. The normal pollen rate was higher in 35S: *CEP1*/*myb2* (61.24%, 237 of 387), 35S: *βVPE*/*myb2* (70.92%, 217 of 306) and 35S: *CEP1* + 35S: *βVPE*/*myb2* (83.74%, 273 of 326) than that in *myb2* (39.18%, 125 of 319) (Table 1). The pollen germination rate increased in 35S: *CEP1*/*myb2* (40.67% ± 2.49), 35S: *βVPE*/*myb2* (54.00% ± 3.27) and 35S: *CEP1* + 35S: *βVPE*/*myb2* (79.75% ± 2.15) compared with that in the *myb2* (29.55% ± 2.10) mutant (Table 1). These results show that the increased proteases level in the different complementation transgenic lines rescued the abnormal anther phenotype, indicating that the deficiency of cysteine proteases accounted for the *myb2* mutant phenotype. Taken together, MYB2 affects tapetal PCD and pollen development by directly regulating expression of the proteases *CEP1* and *βVPE*.

## 3. Discussion

### 3.1. MYB2 Is a Key Component during Tapetal PCD and Pollen Development

*MYB2* has mostly been described to function in salt and drought stress responses, ABA signaling, and plant senescence in previous studies. For instance, MYB2 directly activates the expression of *miR399f*, which modulates ABA, salt, drought and phosphate starvation responses [32,33]. *MYB2* also functions in plant senescence by controlling cytokinin production and axillary bud outgrowth [34]. Previous studies have reported that *MYB2* is expressed in anthers but no further reports are available about its function during the anther development [29]. In our study, the spatiotemporal expression pattern revealed that *MYB2* was highly expressed in tapetum cells from stages 5 to 11. Failed tapetal wall degeneration and a decrease in secretory machinery, including tapetosomes and elaioplasts were shown in the *myb2* mutant. The pollen grains in the *myb2* mutant were shrunk along with abnormal pollen exine. These data provide direct evidence that *MYB2* is essential for normal tapetal PCD and pollen formation.

*AG*, *SPL/NZZ*, *bHLH010*, *bHLH089*, *bHLH091*, *DYT1*, *TDF1*, *AMS*, *MYB80*, and *MS1* form a complex transcription regulatory network that regulates tapetal development in *Arabidopsis thaliana* [16,35,36,37,38,39,40]. We found no significant difference (fold change > 2) in *MYB2* expression in the *spl/nzz*, *dyt1*, *tdf1*, *ams*, *myb80*, or *ms1* mutants, suggesting that *MYB2* is not regulated by these transcription factors [20,28,38,41,42,43]. The expression of these transcription factors and *MYB2* during different stages of anther development was analyzed. *MYB80* (expressed at stages 5–9), *AMS* (appeared at stage 5), and *MS1* (appeared at stage 7) are likely regulated by *MYB2*, as their expression stages overlapped with the *MYB2* gene [21,38,44]. In addition, the promoter sequence analyzed with the online program PLACE suggested that at least one type of MYB2 cis-element was observed in the *MYB80*, *AMS*, and *MS1* promoter (Supplement Table S2). Based on this analysis, *MYB80*, *AMS*, and *MS1* are proposed to be downstream of *MYB2*. However, the transcription factors that regulate the expression of *MYB2* deserve further investigation.

*MYB2* encodes a transcription activator containing R2R3 MYB domains in *Arabidopsis thaliana* [34]. Several *Arabidopsis* MYB family transcription factors are expressed in the anthers, such as *MYB26*, *MYB33/65*, *MYB32*, *MYB35* (also named *TDF1*), *MYB99*, and *MYB80*. *MYB26* regulates secondary thickening in the anther endothecium and is critical for dehiscence of the anther [45]. *MYB32* and *MYB99* control pollen wall formation by affecting the phenylpropanoid pathway [46,47]. *MYB33/65*, *MYB35*, and *MYB80* play important roles in governing tapetal and pollen development [28,48,49]. *MYB33/65* and *MYB35* function in early tapetal development during meiosis [36,50]. *MYB80* is important for late tapetal development during microgametogenesis [36,38]. In our study, microsporocyte meiosis and microspore release were normal in the *myb2* mutant during the early stage of tapetal development. Nevertheless, defective phenotypes of the *myb2* mutant were observed, as the tapetal wall remained intact, secretion visibly decreased, and pollen was aborted. These results suggest that *MYB2* may acts as a late regulator in tapetal PCD and pollen development. This may explain the presence of some normal pollen grains (29.55% ± 2.10) in *myb2* mutants. Both *MYB80* and *MYB2* are associated with late tapetum degradation, and whether *MYB2* functions upstream of *MYB80* in tapetal development needs further investigation.

### 3.2. MYB2 Directly Regulates Expression of the Protease to Supervise Tapetal PCD

Previous studies have revealed that the cysteine protease CEP1 is an irreplaceable executor involved in tapetum cell PCD and pollen formation. The vacuolar processing enzyme βVPE is indirectly involved in this process by activating the maturation of CEP1 [14,15]. In this study, the EMSA and dual-luciferase assay revealed that MYB2 regulated expression of the *CEP1* and *βVPE* genes by directly binding to their promoter sequence, resulting in decreased *CEP1* and *βVPE* expression in the *myb2* mutant. However, the *myb2* phenotype was restored in different proteases complementation transgenic lines. These results suggest that *MYB2* is involved in tapetal PCD and pollen development by directly activating *CEP1* and *βVPE* expression.

Many papain-like proteases are synthesized as inactive preproprotein with a signal peptide and an auto-inhibitory prodomain and require a proteolytic process to form mature functional enzymes [51,52]. CEP1 transforms into its mature form in two ways: self-catalytically and by activation of βVPE [15]. Thus, in the 35S: *CEP1*/*myb2* complementation transgenic line, a few CEP1 precursors possibly transformed into mature enzymes self-catalytically even in the absence of βVPE, which contribute to partly rescue of the *myb2* phenotype. In addition, the degree of recovery of 35S: *βVPE*/*myb2* was higher than 35S: *CEP1*/*myb2*, in agreement with our previous study suggesting that βVPE acts as a trigger in the protease catalytic cascade and that other proteases besides CEP1 are activated by βVPE [15].

The proteases including RD19A, RD19C, RD21A, CP51, and UNDEAD are speculated to function in tapetal degeneration [12,20,28]. The *MYB2* regulatory elements were analyzed in their promoter sequence using the online program PLACE. At least one type of *MYB2* cis-element sequences was observed in *RD19A*, *RD19C*, *RD21A*, *CP51*, and the *UNDEAD* promoter, suggesting that those proteases were possible target genes of MYB2 ( Supplemental Table S2). This may explain why the overexpression both *CEP1* and *βVPE* genes in the *myb2* background did not fully rescue the *myb2* phenotype.

**Figure 8:**
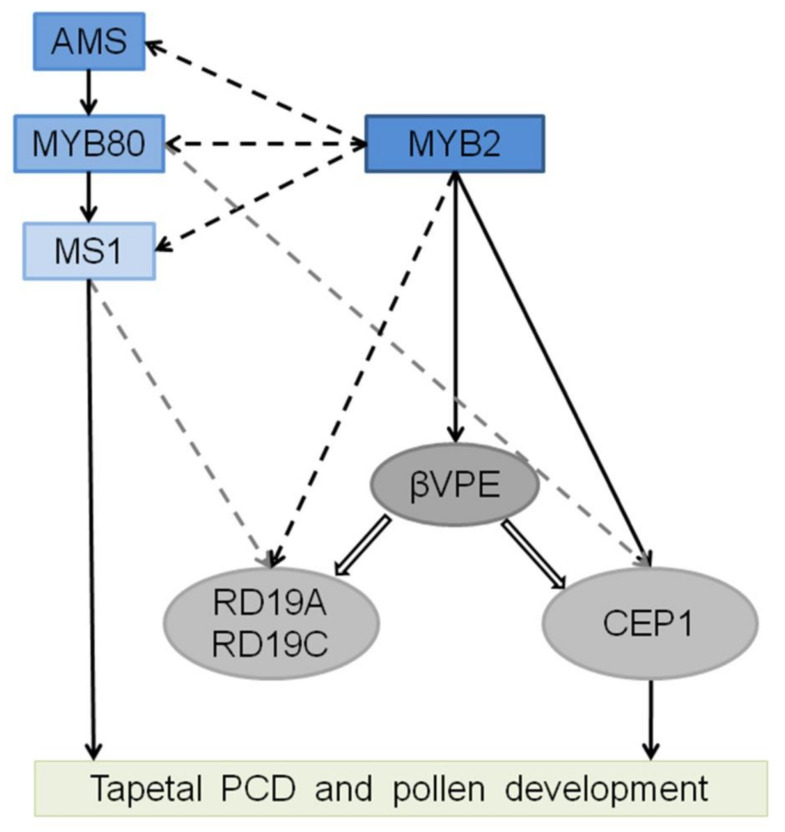
The regulatory model of the *MYB2* during tapetum PCD and pollen development. The rectangles represent transcription factors and the ovals represent proteases. The direct regulation confirmed by the experiment is represented by solid black arrows. The cis-regulatory element analysis is represented by dashed black arrows. The probably regulatory downstream based on microarray data is represented by gray dashed arrows. Proteases activation confirmed by the experiment is represented by hollow arrows.

Vacuolar processing enzymes (VPEs) are cysteine proteases and responsible for the maturation and activation of vacuolar proteins during plant development and immunity [53,54]. VPEs are synthesized as inactive precursors, from which they are self-catalytically activated by sequential removal of the C-terminal and N-terminal propeptides to be converted into mature enzymes under acidic conditions [54,55]. *βVPE* encodes a VPE and the pro-βVPE is transformed to mature form by self-catalytically at stages 5–8 during another development. The maturation of other vacuolar proteases partly or completely relies on the activation of βVPE before rupture of the vacuole [15]. The vacuole begins to be acidified at stage 6 during tapetal development and is completely degraded by late stage 8. A model of *MYB2* function is presented based on the above analysis. Before the acidification of the vacuole, *CEP1*, *βVPE*, *RD19A*, *RD19C*, *RD21A*, *CP51*, and *UNDEAD* expression were directly induced by MYB2 to synthesize inactive proprotein precursors. Pro-βVPE transformed into mature enzyme after the vacuole acidification, which acted as the trigger to activate other vacuolar proteins (CEP1, RD19A, and RD19C) and transform the inactive proprotein into mature form by the activation of βVPE [15]. After rupture of the vacuole, βVPE released into the cytoplasm and rapidly degraded and other proteases acted as executors to participate in tapetal degeneration, except for UNDEAD, which is located in mitochondria and may hydrolyze an apoptosis-inducing protein in the mitochondria that participates in PCD [28]. MYB2 plays a crucial role in regulating the expression of proteases, which ensures that activation of proteases causes a proteolytic cascade resulting in tapetal PCD. Based on our results and the previous transcription-regulating network, we established a model of MYB2-related transcription factor/proteases regulatory and activative cascade (Figure 8).

## 4. Materials and Methods

### 4.1. Plant Materials and Growth Conditions

*Arabidopsis thaliana* accession Columbia (Col-0) was used as the wild-type control. All plants were grown in a soil mixture (5:3:2 mixture of peat moss-enriched soil: vermiculite: perlite) with a 16-h light and 8-h dark photoperiod at 23 °C. Homozygous T-DNA insertion mutants were identified by PCR using MYB2-BP/LP/RP primers ( Supplemental Table S1).

### 4.2. Characterization of the Plants Phenotype

Germination was assessed by culturing fresh pollen grains of different transgenic plants in germination medium (pH 5.8–6.0) containing 1% (*w*/*v*) agar, 15% (*w*/*v*) sucrose, 3 mM CaCl_2_, 1 mM H_3_BO_3_, and 56 mM inositol (Sigma-Aldrich, St. Louis, MO, USA) at 20 °C for 4 h. At least 100 pollen grains were counted for each group. Each group was repeated three times with wild type and different transgenic plants. Arabidopsis pollen germination images were acquired using an M165C microscope (Leica, Wentzler, Germany).

### 4.3. Semi-Thin Sections

Anthers from wild type and mutants at various development stages were fixed in glutaraldehyde fixation solution (2.5% glutaraldehyde, 0.1 M PBS, pH 7.4) for 12 h before being dehydrated in an alcohol gradient series (30 min each at 50%, 70%, 95%, and 100% alcohol) to prepare semi-thin sections. The samples were sequentially embedded in low viscosity Spurr resin at 70 °C for 12 h. Semi-thin sections of 800 nm were cut using a UC6 ultramicrotome (Leica, Wentzler, Germany) stained with 1% toluidine blue O (Sigma-Aldrich, St. Louis, MO, USA) and photographed using a Leica DM 6000 B microscope (Leica, Wentzler, Germany).

### 4.4. TEM

The anther were collected from both wild-type and mutant plants and fixed in glutaraldehyde fixation solution (2.5% Glutaraldehyde, 0.1 M PBS, pH 7.4) for 12 h before being dehydrated in an alcohol gradient series (30 min each at 50%, 70%, 95%, and 100% alcohol) to prepare for TEM. The samples were sequentially embedded in low-viscosity Spurr resin at 70 °C for 12 h. Ultrathin sections (70 nm) were obtained with a UC6 ultramicrotome (Leica, Wentzler, Germany) and then double-stained with 2% (*w*/*v*) uranyl acetate and 2.6% (*w*/*v*) lead citrate aqueous solution. Observations were made and images were captured with an H-7650 transmission electron microscope (Hitachi, Tokyo, Japan) at 80 KV and an 832 charge-coupled device camera (Panasonic, Japan).

### 4.5. Scanning Electron Microscopy

Freshly desiccated pollen grains were collected from wild-type, *myb2* mutants, and different transgenic plants and mounted on SEM stubs. The mounted samples were coated with palladium-gold in a sputter coater (E-1010; Hitachi, Tokyo, Japan) and observed and imaged by SEM (S-3400N, Hitachi,Tokyo, Japan) at an acceleration voltage of 10 kV. For each line, pollen grains from 6 independent plants were collected for SEM and at least 300 pollen grains were counted for normal pollen rate.

### 4.6. TUNEL

Wild type and mutant buds at different stages were fixed in polyoxymethylene and glutaraldehyde fixation solution (4% polyoxymethylene, 2.5% glutaraldehyde) at 4 °C for 24 h. The samples were dehydrated through an alcohol gradient series (30 min each at 50%, 70%, 95%, and 100% alcohol) and cleared in a xylene/alcohol gradient series (1 h each at 70%, 85%, 90% and 100% xylene). The samples were incubated in xylene/paraffin (1:1) overnight at 38 °C and dipped in 58 °C paraffin three times daily. The 8-μM Parafilm sections of treated buds were assessed with the TUNEL apoptosis detection kit (DeadEnd Fluorometic TUNEL system; Promega, Madison, WI, USA) according to the manufacturer’s instructions. The samples were observed using a Leica DMI6000CS confocal laser scanning microscope (Leica, Wentzler, Germany).

### 4.7. GUS Assay

The buds from the transgenic lines were treated with 90% (v/v) pre-cooled acetone for 1 h, subsequently stained with X-Gluc solution (Sigma-Aldrich, St. Louis, MO, USA), and incubated at 37 °C for 12 h to visualize GUS activity. The buds were fixed in formaldehyde-acetic acid-ethanol for 24 h at 4 °C. The bud samples were dehydrated and cleared in an alcohol gradient series (30 min each at 50%, 70%, 95%, and 100% alcohol) and xylene/alcohol gradient series (1 h each at 70%, 85%, 90%, and 100% xylene) respectively before being dipped in paraffin at 62 °C. The bud-paraffin was cut into 8-μM sections using a UC6 ultramicrotome (Leica, Wentzler, Germany). The parafilm sections of the treated buds were observed under an M165C microscope (Leica, Wentzler, Germany).

### 4.8. In Situ Hybridization

A *MYB2* cDNA fragment was amplified by PCR with the MYB2-specific primers ( Supplemental Table S1) and then inserted into the pSPT19 plasmid (Kelei-bio, Shanghai, China) to construct a plasmid that was used to synthesize the antisense and sense probes. Subsequently, the probes were generated using the DIGRNA labeling kit (Roche Diagnostics, Indianapolis, IN, USA) according to the manufacturer’s instructions. The anthers were fixed and hybridized and the hybridized probes were detected and images were obtained using a Leica DM6000 B microscope (Leica, Wentzler, Germany) according to the protocol of Zhang et al. [14].

### 4.9. RNA Extraction and RT-qPCR Analysis

Total RNA was extracted and cDNA was synthesized was performed according to the instructions for the plant total RNA extraction kit (Aidlab, Beijing, China) and the Fastking RT Kit (TIANGEN, Beijing, China). The RT-qPCR analyses were performed using SYBR Green qPCR Mix (TIANGEN) on an iQ5 Multicolor Real-Time PCR detection system (Bio-Rad Laboratories, Hercules, CA, USA). The primers used for RT-qPCR are shown in Supplemental Table S1 and *Arabidopsis ACTIN* gene (AT2G37620) was used as the internal control. The PCR conditions were 94 °C for 3 min, 40 cycles at 94 °C for 10 s, 55 °C for 20 s, 72 °C for 20 s, and 60 °C for 30 s, and 72 °C for 1 min. All reactions were run in triplicate for each sample. Data were analyzed using the iQ5 (Bio-Rad) software (Bio-Rad Laboratories, Hercules, CA, USA), and the difference in gene expression was calculated using the 2^-ΔΔ^Ct method. Data are means (±SD) of three replicates (Supplemental Data Set S1).

### 4.10. EMSA

The CDS encoding full-length *MYB2* (822 bp) fused with His protein tag was expressed in Rosetta (DE3) using pET-30a. The HIS-MYB2 fusion proteins were obtained from prokaryotic expression induced with 0.3 mM IPTG at 24 °C for 15 min. The recombinant MYB2 was purified by Ni-NTA and eluted with gradient series imidazole buffer. The *CEP1* promoter was divided into nine fragments by PCR using specific primers and labeled by biotin using EMSA Probe Biotin Labeling Kit (Beyontime Biotechnology, Shanghai, China) ( Supplemental Table S1). The short probes of the *CEP1* and *βVPE* promoters were shown in specific primers ( Supplemental Table S1) and synthesized by Sangon Biotech (Beijing, China). The EMSA assay was performed according to the manufacturer’s protocols with the EMSA Probe Biotin Labeling Kit and Chemiluminescent EMSA Kit (Beyontime Biotechnology, Shanghai, China). Briefly, biotin-labeled probes and fusion proteins were mixed in a binding buffer for 30 min at 25 °C. The HIS protein was used as a negative control.

### 4.11. Transactivation Assay

The *CEP1* and *βVPE* promoter fragments containing or lacking the MYB2 binding sites were inserted into the pGreenII 0800-LUC vector to generate the reporter constructs. The *MYB2* CDS was inserted into the pGreenII 62-SK vector to generate the 35S: *MYB2* effector plasmid. All gene sequences were amplified by PCR with the specific primers ( Supplemental Table S1). The combination vectors were co-expressed into tobacco leaves. Transactivation was exhibited by the ratio of LUC/REN using Dual-Luciferase Reporter Assay System (Promega, Madison, USA). Experimental procedures of transformation and dual-luciferase (LUC) activity were as described in He et al. [56]. Each group had at least three replicates and data are means (±SD) of three replicates.

### 4.12. Complementation of the myb2 Mutant

The 2666-kb genomic fragment including the promoter and the *MYB2* genomic sequence and *βVPE* CDS were amplified using specific primers ( Supplemental Table S1), and the fragments were cloned into the pCAMBIAI1301 vector to generate Pro*MYB2*: *MYB2*, 35S: *βVPE* for the functional complementation test. The *CEP1* CDS was amplified using specific primers ( Supplemental Table S1) and inserted into the pBI121 for the CEP1 functional complementation test. The 35S: *βVPE*-Nos fragment were amplified from the above pCAMBIAI1301-*βVPE* vector and inserted into the pBI121-CEP1 vector to generate 35S: *CEP1*-Nos and 35S: *βVPE*-Nos for the CEP1 and *βVPE* functional complementation test. These four constructs were introduced individually into the *myb2* mutant background using the floral dip method. All transgenic lines were screened on solid 1/2 MS medium containing Hygromycin or Kanamycin (Sigma-Aldrich, St. Louis, MO, USA) and positive lines were transferred to soil used for the phenotypic observation.

## 5. Conclusions

In summary, *MYB2* is involved in tapetal PCD and pollen development by directly regulating expression of the protease *CEP1* and *βVPE*. This study not only determined the function of MYB2 in tapetal PCD and pollen development but also revealed a MYB2-related transcription factor/proteases regulatory and activative cascade in this process.

## Figures and Tables

**Table 1:** The analysis of normal pollen rate and pollen germination rate in different plants.

Plants	Normal Pollen Rate	Pollen Germination Rate
WT	92.96% (317/341)	87.50% ± 1.68
*myb2*	39.18% (125/319)	29.55% ± 2.10
35S: *CEP1*/*myb2*	61.24% (237/387)	40.67% ± 2.49
35S: *βVPE*/*myb2*	70.92% (217/306)	54.00% ± 3.27
35S: *CEP1* + 35S: *βVPE*/*myb2*	83.74% (273/326)	79.75% ± 2.15

## Data Availability

The data presented in this study are available on request from the corresponding author.

## Supplementary Materials

The following are available online at www.mdpi.com/ijms1637812/s1.

## Accession Numbers

Sequence data from this article can be found in Arabidopsis Genome Initiative database under accession number AT2G47190 (*MYB2*), AT5G50260 (*CEP1*), AT1G62710 (*βVPE*), AT2G16910 (*AMS*), AT5G56110 (*MYB80*), AT5G22260 (*MS1*), AT4G39090 (*RD19A*), AT1G47128 (*RD21A*), AT4G12920 (*UNDEAD*), AT4G16190 (*RD19C*), and AT1G06260 (*CP51*).
