# Supplementary material for: MYB2 Is Important for Tapetal PCD and Pollen Development by Directly Activating Protease Expression in Arabidopsis
Source: Int J Mol Sci. 2022 Mar 24;23(7):3563. doi: 10.3390/ijms23073563 (PMC8998314; doi:10.3390/ijms23073563)
Supplement: Supplementary file 1 [file ijms-23-03563-s001.zip › Supplemental Materials.pdf]

5’-GGTGAAGAGGAAGAAGAGTGAGATAAAAATAAAAAAATGTTATAATCTGATTAGACTAAAG
TTTAGTCTAATATCTTATATAAACAGTAATCTATTATTGGGCCTTACTTGACCCAATAGGCTTTTA
TATTTCCTTATTCACACCGACCTACTCCACCAAAATTAAGACTATATAGTACACAAAGAGCCGT
TTCTAGCAAGATTTCTTTCCTCGGCCTTATCGCGTCGAATTCAAGCCGGCGAACGAATACAT
TTATTTTTCTGAAACAAGATGGATTATCGGTTACAGATTTTAGAAGCAAACGCAATTCATGACA
CTGCATCCTCTTTGTAGTGATGTGATGTGCGTGTTAGCTGTGTGTGTCCGCGTTTATCTTTTT
TTTTTTTGTTACTTTTCTCCTAACTTAGTTGTTATTACGTGCTTTTCCCAATATCAAGTTAAATC
ACACACACTTCCCACCACCTTCCTTCATTTTCTTTCCGAAATTTAGCATTTTAGTCCTCTTTT
GACAAATAGTATCTTCTTTCTTAAATTTAAGATCTTTCTTTAGTCTTTAGACAACAGTTATTGTA
ATGTCTTTTAGTTGAATGACATAAGTTAGGCCAAAATAATTTGAGTTCAATTTTCAAAAGCGTT
GATGAAAGCTAAAACCAATGACTAAAAATGACATCTAATCGGCTCCTGAAAACCATAATCAGA
TATCATATGACAAATACTAAACCAAAATTTTTACCTTTCAAATAAAATAATATTTGCAGATAGATT
TAAAATAGTAAAGGTTATTGAAAAAAAAATACAATAGAAAAACATAATATTACCTAATATTTATTA
CATTTTAAAAGATATATAGAAAAACAAAATCTTAGTTTCGACGATGGAATTTTTTTTTTTATATAG
AACATCACATACCAAATAACTTTAGTTCTCTTATTGTGAAAACAATGGAAGAAATTGACTTTTT
AAAAATAATTTTGAAATTGATTTCTTTGTTCACATACCAAACAACTTCGTTTTATTATCGTGAAA
AAAAGATAACGTAAATGGTTAGCCACACATGCACGGCTTTTCCAAATTATCATAAGGATCCAT
ATTTGTAAAAGAAAAAAATACATAATCCACATAATTAATCATAGTGAGGTTACATGAATAACAGA
GTTCCTAGCTAGTAAAGATAAATTGTTGCGTACAAGTTGTGCTTTGATTAATTATCACTATACG
TGCTTCATATAAAAATGTCATTTTACACTTATATATATTAAGTCATCTCATCGCCGACTATTTCC
CAAAATCATCAATTATTGTTACCAAGAATAAGAAAAGTAAAATGAGAGAGCTTGCTTGCGCCA
TTAAATCAACATAAAGTTCAATTATAGTTTGAAATCTTTATGAATCTCCATTAATTAATGTATCCA
TGTCACTAACCTTGAAATCTTTGTTGAATCCCACAAGTAATCCTTT-3’

GGT Fragment 1 CAC Fragment 2 GTG Fragment 3 CCA Fragment 4 TCT Fragment 5
CTT Fragment 6 GGT Fragment 7 GTG Fragment 8 CTT Fragment 9

- Supplemental Figure S1. *CEP1* promoter sequence and predicted MYB2 binding site. The *CEP1* promoter was divided to nine fragments and each fragment is represented by different color. The red fronts represent MYB2 binding site.

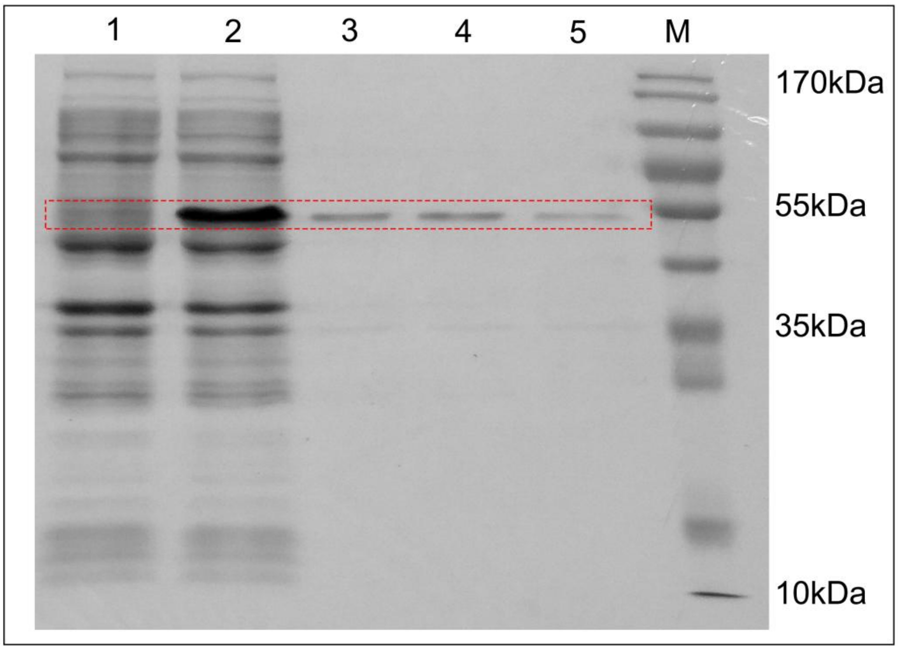

Supplemental Figure S2. Purified recombinant MYB2 used for EMSA.

M: Protein Marker; Lane 1: Negative control; Lane 2: MYB2 prokaryotic expression bacteria proteins after IPTG induction; Lane 3, 4, and 5: Purified recombinant MYB2 protein

Supplemental Table S1. List of primers used in this study

Name	Primers (5′-3′)
qPCR-Actin	F: CGTATGAGCAAAGAGATCAC<br>R: CACATCTGTTGGAAGGTGCT
qPCR-MYB2	F: CGTCCAGATGTTAGAAGAGGC<br>R: GTGGGTAATGATTGGGCGTTG
qPCR-CEP1	F:CTATTGATGCTGGAGGCTCAGACT<br>R:GAATCCCTCTCTGCATTCTTATGT
qPCR-βVPE	F:GATTCTTATGCCGACAGAGG<br>R:CCTGGTGTCTGTAGTTTCCA
LUC-ProCEP1-1	F:TCGAATTCCTGCAGCCCGGGGGTGAAGAGGAAGAAGAGTGAG<br>R:GCTCTAGAACTAGTGGATCCAAAGGATTACTTGTGGGATTC
LUC-ProCEP1-2	F:TCGAATTCCTGCAGCCCGGGCCACCACCTTCCTTCATTTTC<br>R:GCTCTAGAACTAGTGGATCCAAAGGATTACTTGTGGGATTC
LUC-ProCEP1-3	F:TCGAATTCCTGCAGCCCGGGGTTAGCCACACATGCACGGC<br>R:GCTCTAGAACTAGTGGATCCAAAGGATTACTTGTGGGATTC
LUC-ProβVPE-1	F:TCGAATTCCTGCAGCCCGGGAAATGGGGGCTAAAACCCTAGC<br>R:GCTCTAGAACTAGTGGATCCCAATTGTCTAATTATATTTAAT
LUC-ProβVPE-2	F:TCGAATTCCTGCAGCCCGGGTTAGAGCTCTGGGTTAATTA<br>R:GCTCTAGAACTAGTGGATCCCAATTGTCTAATTATATTTAAT
LUC-ProβVPE-3	F:TCGAATTCCTGCAGCCCGGGAGTGTACACGTGTTCGGACT<br>R:GCTCTAGAACTAGTGGATCCCAATTGTCTAATTATATTTAAT
ProMYB2	F:GAATTCTTGTATTTGCTAATCTGTTTTTG<br>R:GGGGTACCAGATTTGAAGTGATTAAGCAATG
MYB2	F:CGGGATCCAATCCACAAAACCATTCACACCATCTC<br>R:GTCGACTTAAGAACTTTTCCTTGGAATGAT
βVPE	F:ATGGCTAAGTCTTGCTATTTC<br>R:TCAGGCGCTATAGCCTAAGATTG

Probe-F1	F:GGTGAAGAGGAAGAAGAG<br>R:GGCTCTTTGTGTACTATATAG
Probe-F2	F:GTACACAAAGAGCCGTTTC<br>R:GGACACACACAGCTAACAC
Probe-F3	F:GTGTGTCCGCGTTTATC<br>R:GTCAAAAGAGGACTAAAATGC
Probe-F4	F:CCACCACCTTCCTTC<br>R:CAGGAGCCGATTAGATG
Probe-F5	F:CATCTAATCGGCTCCTG<br>R:CCATCGTCGAAACTAAG
Probe-F6	F:CTTAGTTTCGACGATGG<br>R:GCATGTGTGGCTAACC
Probe-F7	F:GGTTAGCCACACATGC<br>R:GTGATAATTAATCAAAGCACAAC
Probe-F8	F:GTTGTGCTTTGATTAATTATCAC<br>R:GATTTAATGGCGCAAGC
Probe-F9	F:GCTTGCGCCATTAAATC<br>R:AAAGGATTACTTGTGGGATTC
MYB2-BP	F:ATTTTGCCGATTTCGGAAC
MYB2-LP	F:AACACTTATGCCGTGTTCCAG
MYB2-RP	R:GAGAATTCGAAGACGTTGCTG
In situ-MYB2	F:TGGATAGAGACGAAGTTGACTAGGATTGCA<br>R:TCTCGGCATCCAAACATTTCTCATAGTC
35S：βVPE-NOS	F:TGACCATGATTACGCCAAGCTTAGATTAGCCTTTTCAATTTCA<br>R:GGACCTGCAGGCATGCAAGCTTGATCTAGTAACATAGATGAC

Supplemental Table S2. Promoter analysis of transcription factors and protease genes for MYB2 binding sites

Gene	SequenCes(5′-3′)
AMS	(-973)TTACGTAATCATGAATTTCAGCTTCATGAAAGGTGGACA GTTGGGTCTATGGATATTTATTAGGAGACAAAGCTAGCTCAG CTTTTGCTAGTTGCTACGCATAACAATATTTATTTGTCGTTTAT TCTCAACTCAAATATTTGCATATGTCTTCACACAATTATTTCTG TAAATTTTTATCACTCTATAACATATTTTACGTCGGAACTAAAG AATCAAGGAAAACTAAAATCGAATAATAAGAAAATGACAAAGA AGGGAAAATAAATAGTAGTTGAGACACCGAAAGAAGAATGAA TAACCTAAACGCAACGAAGAGAAGCTCTCAACAAAGTTAGTT AGGGTTTTACACGTTATCTTAGCTCATGCAATCTGCCCCTTTC ATCATTCTAATTCTTTTTATAATATATACTT(-629)
MYB80	(-376)AGGAGTTGACCAGGCGTTGAGTGGGTGACCTACCGGC TTAAGGGAATCACACGCTCTGTAGCGTTGAATTCTTTAAAGA GTTTAATCAATTCATGCACATCTGATGCTAATTTTTACTATGTT TTCATTTTTGTTAAATCAATTTTATTATATATATACATACATTTC CTTTCTTCTTTTGCAATTTAAAAGCCTTGATTACAAGAAAGTAC AAAAATCAAAAGTTTGGCAGTTTTAATTCTTTGGGTGGGGCAA TCTTTGATATATATAAGTTTTTTTCATTTCTTAACCAAATTTTCT TTTTTCTCGTTAGTAAATTAAGTAAAGAGTAATCAAATCGATCA AGAGATAAAAACTAGAAAGAAAGAAAGAAGAA(-1)
MS1	(-953)CTGAACTTTTAACTCTTTTTTTTTACGAAGATTGTAACAC TAAAAACATATATTTGAAGCACTGAAGTGACTGTAAGTACCCA AAGTTGTTGTGATCACTGCAATCATTAAGGTGAGTACAATTTT GAATGTAATATATTAACTATTAAGTAAATACCATTGCAATTTGC AAACCACTTTTTCTGAGAACATAATTTGAAACTTTCTTTAAACC GCTAAAAAACATCTATTGATGTAAACACCTTTTTCTATTGATGT

	AACATATTAATTATGTTTAATTAACAATAACTATGTGGGTTTGA ACATATAGTGATGACAAATCATCAACACTATCACAAATCAATA AACTACATATCAATTAAAACATTGATAATCGACTAAATTGAGTT TGTCCAACCACGCAAACACACACGT(-540)
RD19A	(-626)TAAAACGCATATTCAAAGAGACATATTTGATCATCGTGT CTTCATAATTTGGTTCAAAGTACAGCCAAAAACCAGGAGGTA CACTAAAAATCAATAAAATTACATTACAAATTATTTTTAGTACA CTGGGAACATAAATAAATGAATCAGAGAAGAGAAACATGGCA ACTACCGATCATGAGCTTCTACATTTGTCTCAGAAAAAGAGG AAGATAGAACTATTTTTTAAAGTTTTTAGAAAAGAGAGAATTAA TATTATCCAAGACAACACGGCACTGCTAACAACAAGTTGAAC CTATCCCATTCCATTACTCCATACCCATTCAATGATTAACACG TGGTTCTACATCATTGGTTAACTCTCTCCTTTCCCCTTGGATA AGGCATCTTAAAATAATAATAAATACCATAAAT(-212)
RD21A	(-930)GTGGACATTTTATTTTTGGCTTGCCAATTAGAGATCAGT GACGTCTCATCTCTTCCTACCCATATTGTCTAACCAATAATTG ACCATCACATATTCACATTTATAGATTTCATCTTTACACTATTG TTTTTTTTTTCACTTATCAAAATATCTCAACCGACCCTGAAATT AATTAGGATTTTTAAATCGGATTTTGGCTCAAGTTTTTGTTTTT GAATATTTAAGAAAATAAATACAATTAAGTATAAGTTTAGAGAT GATTTGTTCATTTATTTGAAGTTTTTGATTTTTTTTTTCTTATTA AAATGACAAAAAGAATGATTTGAGTTCTTTTCTTTTTTGGCTCA AGATTTATTTTGGCCCACTTTTCTTTTCAATATTATTAAGAAAA CTTGACTCATAAATTTTTGTTGGGAAAAAGAGGAAATGAGAAA AGGGATAATAATAACAAACAAAAAAATGATTTGATGTTGGTTT GGGTTC(-448)
UNDEAD	(-581)CCTACATCAAACCAAACTATATCCTATATACATTACCAA CAAGAATTTCTATTAACTAACAAACTTCTTCTATCTTTCCTCAC

	TATTTAACCATTAAACCAACACATCTCTTACTTATACAATTAAA TTAACTTATTATGAAGAACCACTAAACCAACTTGTACCCTTATT TGATATGATATTAGATGATCTACATATTTTATTATTACATACTC AAAATCCCCACATCAAACCAAACTATATCCCAACATACATTAC CAATAAGAATTTCTACTAACTAACCTACTTCTTCTTTCTTCCCT CACTCTTTAACCATTAAACCAACTTTTCTCTTACTTTAGTGTCA AACTACAACTTCACAAATCTTCCATTCACTATTATAATATCACC ACATCTTAAAAAACAAATCACATCCTTAATTAATAACTCTAAAA ACAGTTTAA(-1)
RD19C	(-215)CCACTCCTCTTTGCTGTTGCAATTCGGCAATCGTTCTAG ATATGGTTTCCATTTCAAATCATGATATGCATTGACTTTTTCCA TGTGGCGTTCGGAAATCTTTCATCTATACTACGTCTACGTTGC AAGTTTTGCAAAATGTTTAAATTAGTAGAATCTCACGTATATAA AAACTTTAGTCGCCAAATTGAAAATGGAGAATGAATGGTAAAC TA(-1)
CP51	(-301)ACCAGTTTTACAACGAGTACCTTTACCTGCAAAATGTCA AAAGAAGAGAAAATTTCAGTTTTCAAGACCAAAAAAAAAATGA CTTCTGGAGAAACAGGCATAACATAAACACATATGTCAGTTG ACTAAAACAAACTAAATTATACGACTCTCTTAAACTTACGTTTC TGCATATTGTTAAGGATGGGACAGGAGGAAACTTGTCCCACT GGAACTGCAACAAAACACAAAACCTGTGACCAAGAAAGCAGT ACAGGACTGAAGCTTATATACCAGAAACCAAACTCATTTCCCA AACTCC(-1)

MYB2 binding sites: A/TAACCA C/TAACG/TG

Minus signs in position indicate that the nucleotide exists upstream of the 5 terminus of the putative transcription start site. Numbers indicate the nucleotide beginning at the 5 terminus of the cDNA sequences.
